# Knowledge of cervical cancer prevention and HPV vaccination: A survey among 961 college students in Hangzhou, China

**DOI:** 10.1097/MD.0000000000044069

**Published:** 2025-08-22

**Authors:** Jian Zou, Jian-Ying Xu, Xiao-Ping Hua, Zhu Cao, Jing Ye, Yang Li

**Affiliations:** a The Department of Gynecologic Oncology, Women’s Hospital, Zhejiang University School of Medicine, Hangzhou, Zhejiang Province, China; b The Department of Zhejiang Provincial Key Laboratory of Precision Diagnosis and Therapy for Major Gynecological Diseases, Women’s Hospital, Zhejiang University School of Medicine, Hangzhou, Zhejiang Province, China.

**Keywords:** cervical cancer prevention, college students, HPV vaccine, willingness to vaccination

## Abstract

The human papillomavirus (HPV) vaccine is the primary prevention for cervical cancer, which has not yet been added to the planned immunization in our country. However, the attitude and perceptions of college students towards HPV vaccination are unknown. We investigated the knowledge of and attitude towards HPV vaccination and cervical cancer among college students aged 18 years and older. A total of 961 college students in Hangzhou City, Zhejiang Province voluntarily participated in our study by filling out a self-administered questionnaire on HPV vaccination and knowledge of cervical cancer prevention. The age of all the college students was 24.69 ± 4.59 years, including 394 males (41%) and 567 females (59%). A logistic regression model was used to analyze the factors influencing the injection of the HPV vaccine among 961 college students. A total of 96.15% (924/961) of the college students said they had heard of the HPV vaccine, 84.60% (813/961) of the college students were willing to receive the HPV vaccine, and 56.08% (318/567) of the female college students had HPV vaccination. Logistic regression analysis showed that major, educational level, place of household registration, and mother’s education significantly influenced HPV vaccination (*P* < .05). Increasing the knowledge and the coverage of HPV vaccine is important for cervical cancer prevention. Adding courses on the HPV vaccine in high schools and colleges, increasing HPV vaccine consultation clinics in community hospitals, developing more HPV vaccines and reducing the costs may be effective measures.

## 1. Introduction

Globally, cervical cancer is the fourth most common cancer in women, with around 6,60,000 new cases in 2022.^[1]^ And about 94% of the 3,50,000 deaths caused by cervical cancer occurred in low- and middle-income countries. An estimated 1,50,000 incidences and 56,000 deaths occurred in China in 2022.^[2]^ Human papillomavirus (HPV) infection has been identified as a precursor to cervical cancer. WHO’s Global Strategy to Accelerate the Elimination of Cervical Cancer contains 3 key steps: vaccination, screening and treatment. Successful implementation of all 3 steps could reduce more than 40% of new cases of the disease and 5 million related deaths by 2050. A document issued by National Health Commission in China on January 20, 2023. The Action Plan for Accelerating the Elimination of Cervical Cancer (2023–2030) aiming to implement the Healthy China 2030 Plan and respond to the World Health Organization’s global strategy for the elimination of cervical cancer. The plan sets a phased target of 50% cervical cancer screening and 90% treatment for women of appropriate age by 2025, and 70% by 2030. Approximately 90% of cervical cancers occur in low-income and middle-income countries that lack screening and HPV vaccination program.^[3]^ High HPV vaccination coverage in girls can lead to cervical cancer elimination in most low-middle-income countries.^[4]^ Many countries have been included in the national immunization program but not yet in our country. HPV vaccination has been associated with a substantially reduced risk of invasive cervical cancer. How to promote vaccination pilots is a big deal to prevent cervical cancer. Many people have poor knowledge of HPV vaccines and cervical cancer prevention. Improving acceptance of HPV vaccines and cervical cancer screening is important for cervical cancer prevention. The attitude and perceptions among young people towards HPV vaccination are still unknown. This study aimed to investigate the attitude to HPV vaccination among college students and the possible factors influencing vaccination among college students in Hangzhou, China.

## 2. Methods

### 2.1. Study population

A total of 961 college students studying at 4 comprehensive universities in Hangzhou City completed a volunteer questionnaire in October, 2023. The target population was college students aged 18 years and older. The exclusion criteria were as follows: incomplete information and severe mental disorder.

### 2.2. Measurement

We designed a questionnaire on the app named in the questionnaire star, which is popular among college students in our country. Students completed an online questionnaire. A pre-survey was conducted before the formal survey and a final survey was conducted after the revision. The contents of the questionnaire included the age, major, educational level, place of household registration, the parents’ educational level, attitudes toward premarital sex, smoking, initial sexual age, number of sexual partners, and the willing to receive the HPV vaccine, “Do you know how to order the HPV vaccine?” “Have you undergone cervical cancer screening, such as HPV messenger RNA detection and Thinprep cytologic test?” “Are there any issues with your screening results for cervical cancer?”

### 2.3. Information collection

A total of 961 college students from Hangzhou City participated in this study. The study protocol was followed prior to the study. The researchers explained the purpose of the survey to participants and answered their questions. All the participants completed the questionnaire on a volunteer basis. The purpose and objective of the study were explained to all the participants. Privacy, confidentiality, and the right to withdraw were ensured.

### 2.4. Statistical analysis

Data were analyzed using the SPSS v22.0 software (IBM Corp., Armonk). Measurement data are presented as mean ± standard deviation ($\bar{x}\pm S$), and categorical variables are presented as frequencies and percentages (%). Pearson’s chi-squared (χ^2^) test was used to determine statistical significance. Statistical significance was set at *P*<.05.

## 3. Result

### 3.1. The characteristics of all the participants

A total of 961 college students (394 males and 567 females) participated in the study, with the age 24.69 ± 4.59 years. In addition, 84.60% (813/961) of the participants were undergraduates. Of the participants, 13.32% (128/961) were master’s students and 2.08% (20/961) were doctoral students. A total of 96.15% (924/961) of the participants had heard of HPV vaccine. Moreover, 84.60% (813/961) of participants were willing to be vaccinated (Table 1).

**Table 1:** The characteristics of all the 961 college students.

Variable	Group	Frequency (n)	Percent
Gender	Male	394	41.00
	Female	567	59.00
Age	24.69 ± 4.59		
Education level	Junior college and undergraduate	813	84.60
	Postgraduate and doctoral student	148	15.40
Major	Medical	380	39.54
	Nonmedical	581	60.46
Place of household registration	Urban	662	68.89
	Rural	299	31.11
Father’s level of education	High school and above	662	68.89
	Below high school	299	31.11
Mother’s level of education	High school and above	599	62.33
	Below high school	362	37.67
Smoking	Yes	185	19.25
	No	776	80.75
Heard of HPV vaccine	Yes	924	96.15
	No	37	3.85
Injection of HPV vaccine	Complete	318	33.09
	Incomplete	643	66.91
willing to inject the HPV vaccine	Yes	813	84.60
	No	148	15.40
Attitude to premarital sex	Support	633	65.87
	Oppose	328	34.13
Had sexual intercourse	Yes	502	52.24
	No	459	47.76
Number of sexual partners	<3	865	90.01
	3 or more	96	9.99
Have done HPV sreening	Yes	98	10.20
	No	863	89.80
The result of HPV screening	Normal	98	10.20
	Abnormal	0	0.00
HPV vaccine knowledge	Yes	840	87.41
	No	121	12.59

HPV = human papillomavirus.

### 3.2. Univariate analysis for HPV vaccination and the knowledge of cervical cancer prevention among 567 female college students in Hangzhou

To investigate the factors associated with HPV vaccination, we focus on the 567 female students who need to be vaccinated. Among them, 92.42% (524/567) were willing to receive the HPV vaccine and 56.08% (318/567) had received the HPV vaccine. A total of 17.28% (98/567) of participants had undergone cervical cancer screening. A total of 90.82% (515/567) of their initial sexual activity was >18 years of age and 45.33% (257/567) had no history of sexual activity. Of these, 7.58% (43/567) had more than 3 sexual partners.

In the univariate analysis, there were many independent variables such as age, major, education level, place of household registration, parents’ level of education, smoking, had sexual intercourse, attitudes toward premarital sex, number of sexual partners, and HPV vaccine knowledge. The results showed that major, educational level, place of household registration, mother’s level of education, attitudes toward premarital sex, had sexual intercourse, and HPV vaccine knowledge had a statistically significant influence on HPV vaccination (*P* < .05; Table 2).

**Table 2:** Univariate analysis for the injection of HPV vaccine among 567 female college students in Hangzhou.

Variable	Group	Complete injection of HPV vaccine	Incomplete injection of HPV vaccine	*P* value	OR	95% CI
Age	25 or younger	201 (45.45%)	149 (26.28%)	.434	0.867	0.617–1.219
	More than 25	117 (20.63%)	100 (17.64%)			
Educational level	Junior college and undergraduate	254 (44.80%)	230 (40.56%)	<.05	3.050	1.773–5.246
	Postgraduate and doctoral student	64 (11.29%)	19 (0.03%)			
Major	Medical	137 (24.16%)	47 (0.08%)	<.05	3.253	2.208–4.792
	Nonmedical	181 (31.92%)	202 (35.63%)			
Place of household registration	Urban	239 (42.15%)	139 (24.51%)	<.05	2.394	1.676–3.420
	Rural	79 (13.93%)	110 (19.40%)			
Father’s level of education	High school and above	218 (38.45%)	160 (28.22%)	.324	1.213	0.854–1.723
	Below high school	100 (17.64%)	89 (15.70%)			
Mother’s level of education	High school and above	201 (35.45%)	127 (22.40%)	<.05	1.650	1.178–2.312
	Below high school	117 (20.63%)	122 (21.52%)			
Smoking	Yes	28 (4.94%)	15 (2.65%)	.264	1.506	0.786–2.886
	No	290 (51.15%)	234 (41.27%)			
Attitude to premarital sex	Support	216 (38.10%)	141 (24.87%)	<.05	0.612	0.437–0.870
	Oppose	102 (17.99%)	108 (19.05%)			
Had sexual intercourse	Yes	186 (32.80%)	124 (21.87%)	<.05	1.420	1.017–1.983
	No	132 (23.28%)	125 (22.05%)			
Number of sexual partners	<3	291 (51.32%)	233 (41.09%)	.425	1.351	0.711–2.567
	3 or more	27 (4.76%)	16 (2.82%)			
HPV vaccine knowledge	Yes	298 (52.56%)	220 (38.80%)	<.05	1.964	1.083–3.564
	No	20 (3.53%)	29 (5.11%)			

CI = confidence interval, HPV = human papillomavirus, OR = odds ratio.

### 3.3. Logistic regression with multiple covariates for HPV vaccination and the knowledge of cervical cancer prevention among 567 female college students in Hangzhou

To analyze the variables influencing factors of HPV vaccination and cervical cancer prevention among female college students in Hangzhou, logistic regression with multiple covariates was performed. The results showed that major, educational level, place of household, and mother’s education significantly influenced HPV vaccination and knowledge of cervical cancer prevention (*P* < .05). Additionally, we found that students with higher educational levels, mothers with higher educational levels, medical specialties, and urban residence registrations had higher HPV vaccination rates and a greater knowledge of cervical cancer prevention (Table 3).

**Table 3:** Logistic regression with multivariate analysis for the injection of HPV vaccine among 567 female college students in Hangzhou.

Variable	Group	*B*	Standard error	Wald	*P* value	OR	95% CI
Major	Medical	1.256	0.225	31.304	<.05	3.513	2.262–5.455
	Non-medical						
Educational level	Junior college and undergraduate	0.917	0.3	9.38	<.05	2.503	1.391–4.503
	Postgraduate and doctoral student						
Place of household registration	Urban	0.722	0.219	10.851	<.05	2.059	1.34–3.164
	Rural						
Mother’s level of education	High school and above	0.552	0.263	4.399	<.05	1.736	1.037–2.907
	Below high school						

CI = confidence interval, HPV = human papillomavirus, OR = odds ratio.

## 4. Discussion

To accelerating the elimination of cervical cancer in China followed the Healthy China 2030 Plan and respond to the World Health Organization’s global strategy, tertiary prevention measures for cervical cancer need to be further strengthened. The first step is HPV vaccination. HPV vaccination lagged for more than 10 years until the China Food and Drug Administration approved a bivalent HPV vaccine in 2017, and it has not yet been included in the national immunization plan.^[5]^ How to increase the willingness of women to receive HPV vaccine is very important. The attitude and perceptions of young people is unknown. So we focus on the college students in Hangzhou to investigate their willing to HPV vaccination.

In our study, 56.08% (318/567) of female college students received the HPV vaccine. Increasing the coverage of HPV vaccines is essential for cervical cancer prevention. Our study showed that major, educational level, household location, and mother’s education significantly influenced HPV vaccination. This means that the more information about the knowledge of cervical cancer and the HPV vaccine they receive, the higher is the probability of vaccination. A previous study showed that most male participants had poor knowledge of cervical cancer and HPV, even those with a personal or family history of cervical cancer.^[6]^ Another study showed that 87.9% of 800 college students in Zhengzhou City, Northern China, had heard of cervical cancer. Seventy four percentage of them had heard of the HPV vaccine, and only 46.7% had good knowledge of cervical cancer prevention.^[7]^

Currently, there are 3 types of HPV prophylactic vaccines: Bivalent, quadrivalent, and 9-valent HPV vaccines, which are effective in protecting against 90% of HPV infection.^[8–12]^ HPV vaccination and twice-lifetime screening are necessary to achieve cervical cancer elimination in low-to-middle-income countries with more than 90% HPV vaccination coverage and 90% screening uptake.^[4,13]^ An observational study in England showed that the HPV vaccine can reduce the rates of cin3 and cervical cancer, especially in individuals who were offered the vaccine at age 12 to 13 years.^[14]^ Another study in Japan showed that many deaths from cervical cancer could be prevented if vaccination coverage with extended catchup could be rapidly restored.^[15]^ Also, domestic HPV vaccine price and economic benefit are factors to be considered. In 2019, various combined screening and vaccination strategies would incur an additional cost of US$61,57,000–2,21,46,000 and result in 691 to 970 quality-adjusted life-years in China. Strategies that combined screening and vaccination would be more cost-effective than screening alone strategies when the vaccination cost was <$50 for 2 doses.^[16]^

Increasing HPV vaccine coverage is important for the prevention of cervical cancer. It will be useful to strengthen awareness and willingness to receive HPV vaccination, and improve knowledge of cervical cancer prevention. Suppose the mother has a greater understanding of cervical cancer and HPV vaccine, and a higher rate of their daughter getting vaccinated. Improving women’s knowledge about cervical cancer prevention is important. Students mainly understand HPV vaccines and cervical cancer through the media, social publicity, family members, or friends.^[17–19]^ Increasing the courses of HPV vaccines and cervical cancer in college, high school, and middle school may be beneficial in raising public awareness of HPV vaccines and cervical cancer.^[20–22]^ Also, increasing HPV vaccine consultation clinics in community hospitals can help women to have more professional guidance.

How can the coverage of HPV vaccination be improved^[23]^? First, the HPV vaccine is added to the national immunization plan as soon as possible; second, the course of cervical cancer prevention in college, high school, and middle school to increase the knowledge of HPV vaccine and cervical cancer, creating health information memes and infographics to the instagram^[24]^; and third, developing more HPV vaccines and reduce the costs, then more women can afford the cost.

The study was limited to the college students. Women in different fields need to be attention. The data on women in different cities in the whole country need to be investigated to increase the coverage of the HPV vaccine.

## 5. Conclusion

Adding courses on the HPV vaccine in high schools and colleges, increasing HPV vaccine consultation clinics in community hospitals, developing more HPV vaccines and reducing the costs may be effective measures to increase the coverage of HPV vaccine to prevent cervical cancer.

## Acknowledgments

The authors thank the patients for their participation and dedication to the questionnaire survey. We also thank the study group for answering the questions and collecting information.

## Author contributions

**Conceptualization:** Xiao-Ping Hua, Zhu Cao, Jing Ye.

** Data curation:** Jian Zou, Xiao-Ping Hua.

**Formal analysis:** Jian-Ying Xu, Yang Li.

**Software:** Jian-Ying Xu.

**Supervision:** Yang Li.

**Writing – original draft:** Jian Zou.

**Writing – review & editing:** Zhu Cao, Jing Ye.
